# Oligometastasis and oligo-recurrence: more than a mirage

**DOI:** 10.1186/s13014-014-0230-6

**Published:** 2014-10-31

**Authors:** Fang Huang, Gang Wu, Kunyu Yang

**Affiliations:** Wuhan Union Hospital, TongJi Medical College, Huazhong University of Science and Technology, Wuhan, 430022 Hubei China

**Keywords:** Oligometastasis, Oligo-recurrence, Surgery, SBRT, Survival

## Abstract

The standard treatment choice for cancer metastasis has been systemic management, including cytotoxic chemotherapy, hormonal manipulation, and targeted therapy. Emerging evidence has shown an oligometastatic state, an intermediate state between limited primary cancer and polymetastatic cancer, in which local therapy for metastatic lesions results in satisfactory survival comparable to non-metastatic disease. We provide a comprehensive introduction of evidence from experimental and clinical studies in favor of the oligometastatic phenotype, we review the efficacy and safety of surgery and stereotactic body radiotherapy in the treatment of oligometastases, and finally, we discuss the way to differentiate the oligometastatic state from polymetastasis.

## Introduction

Cancer is widely regarded as a systemic disease. Previously, cancer with metastasis has been considered stage IV, an end-stage disease, with the goal of palliative management. The standard treatment choice for metastatic cancer has been systemic management, including cytotoxic chemotherapy, hormonal manipulation, and targeted therapy. Despite recent advances in systemic therapy, prognosis remains poor. Within the population of cancer metastasis, emerging evidence has shown that a fraction of patients have an oligometastatic state, in which local therapy for metastatic lesions results in satisfactory survival comparable to non-metastatic disease. The concept of an oligometastatic state was first proposed by Hellman *et al.* as an intermediate state (≤5 metastases) between limited primary and polymetastatic cancers in which local therapy could achieve long-term survival or cure, with no restrictions on primary lesions [1,2]. In 2006, the concept of oligo-recurrence was defined by Niibe *et al.* as the state that cancer patients have ≤5 metastatic or recurrent lesions with controlled primary lesions [3]. Recently, the concept of sync-oligometastasis was proposed as the state that cancer patients have ≤5 metastatic or recurrent lesions with active primary lesions [4]. The major difference among oligometastasis, oligo-recurrence and sync-oligometastasis was the status of the primary lesion, which is the most important prognostic factor of oligometastasis, and oligo-recurrence showed better prognosis compared with sync-oligometastasis.

Despite the accumulating knowledge on this state, the existence of oligometastasis remains debatable. Although oligometastatic clones are not identified directly, some clonal areas with modest metastatic capacities might lead to oligometastasis, at least in a fraction of all metastatic cases. During the past decades, a large amount of data has shown excellent 5-year survival rates after aggressive local treatment of metastatic disease for many patients, including those with a limited number of metastases, certain primary tumor types, and early T- and N-stage primary tumors. However, many oncologists believe oligometastasis is more like a mirage than a reality. In this review, we provide a comprehensive introduction of evidence favoring the oligometastatic phenotype, we review the efficacy and safety of two methods to treat metastatic lesions [surgery and stereotactic body radiotherapy (SBRT)], and finally, we discuss the manner in which oligometastasis can be differentiated from polymetastasis.

## Review

### Biological basis of the oligometastatic phenotype

Metastasis occurs when genetically unstable cancer cells colonize a tissue microenvironment distant from the primary tumor. With advances in genomic research techniques, including next-generation sequencing and high-resolution genome-wide SNP and copy number analyses, emerging evidence indicates that clones with selective advantages within the primary tumor give rise to distant metastasis. Navin *et al.* applied single-nucleus sequencing to investigate the genetic relationship between a primary breast cancer tumor and its liver metastasis [5], they found that copy number profiles from the primary tumor were highly similar to those from the metastases, indicating that metastatic cells emerge from a main advanced expansion rather than from an earlier intermediate or a completely different subpopulation.

Primary tumors are composed of heterogeneous cell populations, and evidence over the years has shown that tumor clones are not equally able to metastasize. Fidler *et al.* identified a wide range of metastatic ability of B16F1 melanoma cells to colonize the lung [6], supporting the notion of clonal heterogeneity within the primary tumor. Various cell lines with high and low metastatic potential have also been reported in PC-14 human lung adenocarcinoma cells [7] and MHCC97 hepatocellular carcinoma cells [8], and these results have been expanded upon by investigators using KHT sarcoma cells [9].

Further important evidence implying the existence of oligometastasis was proposed by Yachida *et al.* who used next-generation sequencing techniques to analyze the genomes of seven patients with pancreatic cancer metastases in order to evaluate the clonal relationships between the primary and metastatic cancers [10]. Quantitative analysis of the genetic evolution of pancreatic cancer indicated at least a decade between the initiating mutation and the birth of the cancer cell, another five years were required for acquisition of metastatic ability. This temporal nature emphasizes that oligometastastic clones might develop before polymetastatic clones during tumorigenesis.

During this time, intensive research on metastasis-regulating genes has also advanced. Although the genes responsible for the oligometastastic phenotype remain elusive, DNA array analysis has provided important information to distinguish oligometastasis from polymetastasis. Wuttig *et al.* used samples from patients with renal cell carcinoma to identify genes that characterized ‘few’ (<8) or ‘many’ (>16) pulmonary metastases [11]. Analysis of fresh samples from resection of pulmonary metastases revealed 135 genes that were differentially expressed between the ‘few’ and ‘many’ metastasis groups. Furthermore, polymetastatic tumors were enriched by genes that regulate the cell cycle. Based on a meta-analysis of these data and previously published data, an 11-gene classifier was established to predict the number of metastases in patients with renal cell carcinoma. These data provide evidence at the molecular level for the existence of an oligometastastic state, but further work is essential to clarify mechanisms that generate the oligometastastic phenotype.

### Clinical supporting evidence and treatment options for oligometastases

#### Liver oligometastases

The liver is frequently involved in cancer metastasis, especially in cancers from the gastrointestinal tract. This susceptibility is attributed to venous drainage of the gastrointestinal tract via the portal vein and to the liver-specific microenvironment suitable for colonization by certain types of tumor. Local therapy, including surgery and SBRT of liver metastatic lesions, has significantly improved survival in these patients. Much evidence exists showing the efficacy of surgical resection of hepatic metastases from colorectal cancer, with 10-year overall survival (OS) rates of 17-28%, which is far better than those of patients treated with systemic therapy [12].

Long-term survival after resection for non-colorectal cancer liver metastases has also been documented. A systematic review of >1000 patients with breast cancer and liver metastasis showed 2-, 3- and 5-year survival rates of 58-86%, 35-79% and 21-61%, respectively [13]. Liver metastasectomy of neuroendocrine tumors (NET) has also been shown to benefit most patients (95%) and prolong survival [14]. A systematic review of NET liver metastasectomy reported median 5- and 10-year OS rates of 70.5% and 42%, respectively, and median 1-, 3- and 5-year progression free survival (PFS) rates of 63%, 32% and 29%, respectively, which are much better than those from other tumor origins. Hepatectomy of metastases from melanoma also nearly doubles survival, with a median OS of 14 months after surgery increased to 27 months with R0 resection [15]. Summary of hepatic metastasectomy from selected studies were listed in Table 1.

**Table 1:** **Summary of hepatic metastasectomy from selected studies**

***Primary tumor type***	***Year***	***No. patients***	***5-year survival (%)***	***10-year survival (%)***	***References***
***Noncolorectal***	2005	142	26	/	[16]
***Noncolorectal Nonendocrine liver metastases***	2006	1452	36	23	[17]
***Noncolorectal nonneuroendocrine liver metastases***	2007	360	37	/	[18]
***Breast cancer***	2010	41	48	/	[19]
**Soft-tissue sarcoma**	2009	45	49	/	[20]

Although surgical resection is the optimal choice for patients with liver oligometastases, unfortunately, only some patients are eligible for metastasectomy at diagnosis [21]. It is difficult to eradicate gross tumors using conventional radiotherapeutic techniques without accompanied radiation-induced liver disease because of the relative radiosensitivity of the liver [22]. Stereotactic body radiotherapy allows delivery of ablative doses of radiation to metastatic lesions. The efficacy and tolerability of SBRT for liver metastasis has been confirmed by retrospective studies [23-26] showing local control rates around 80% or higher [27]. At Centre Oscar Lambret, 42 patients with 62 liver metastases were treated with SBRT at 40 Gy in 4 fractions and 45 Gy in 3 fractions. 1- and 2-year local control rates were 90% and 86%, respectively, and 1- and 2-year OS rates were 94% and 48%, respectively. In all, 38% of patients suffered grade 1 or 2 toxicity, and one patient had grade 3 epidermitis. Summary of SBRT for liver metastasis from selected studies were listed in Table 2.

**Table 2:** **Summary of SBRT for liver metastasis from selected studies**

***Primary tumor type***	***Year***	***No. patients***	***Local control***	***Toxicity***	***References***
***Mixed (Most colorectal cancer)***	2001	37	81% at 18 month	not mentioned	[28]
***Mixed***	2006	36	93% at 18 months	one case of grade 3 soft tissue toxicity	[29]
***Mixed (Most colorectal cancer)***	2009	68	1-year local control rate was 71%	9% acute grade 3 toxicities and 1% grade 4 toxicity	[30]
***Mixed (Most colorectal cancer)***	2006	25	1- and 2-year local control rates were 94% and 82%	4 cases of grade ≥3 toxicity	[31]
***Mixed (Most colorectal cancer)***	2011	27	30-, 50-, and 60-Gy cohorts were 56%, 89%, and 100% at 24 months respectively	one case of grade 3 toxicity	[32]
**Mixed**	2009	47	1- and 2-year local control rates were 95% and 92%	actuarial rate of grade ≥3 toxicity was 2%	[33]

Although many reports on SBRT for liver metastases are retrospective, prospective trials have been initiated as well. In phase I trials, maximum tolerated doses of SBRT were determined using a range of therapeutic doses in various fractions. In all, 60 Gy was well tolerated and safe and was commonly used as the therapeutic dose in phase II clinical trials. 1- and 2-year local control rates in prospective studies ranged 71-95% and 82-92%, respectively, with no grade 4–5 liver toxicity and few grade 3 toxicities [28-34]. Preliminary reports from a 2013 phase II trial of 61 patients with 3 or more lesions receiving 75 Gy on 3 consecutive days showed a 94% in-field local response rate at 1-year, median OS of 19 months and actuarial survival of 83.5% at 12 months. Grade 3 late toxicity occurred in one patient, with no grade 3 or higher acute toxicity. SBRT offers an alternative, noninvasive approach for patients with liver metastases. The outcomes of serial studies confirm that it is a promising treatment modality with efficacy and safety, bringing benefit to patients with unresectable lesions.

#### Lung oligometastases

Lung metastasis is also a major cause of cancer death, and the lung is the primary venous drainage organ for the entire body except the gastrointestinal tract. The lung is the most extensively studied organ for cancer microenvironment because of its susceptibility to metastasis. Local therapy for patients with lung oligometastases is not new and significantly prolongs survival. Many retrospective surgical studies have shown that patients with limited metastases can have a long-term survival, indicating that limited metastasis may be eliminated in these patients [35-41]. The particular study by Pastorino et al. (1997) is of great importance [35]. They assessed the long-term results of 5206 cases of lung metastases from different origins treated with surgery included in the International Registry of Lung Metastases. The 5-year OS rate of patients with complete metastasectomy was 36%, but it was only 13% for those with incomplete resection. A prospective study including 1720 patients with pulmonary metastatic melanoma showed that metastasectomy is a strong predictor of survival with the hazard ratio of 0.5 (95% CI: 0.4-0.6) [40]. Summary of pulmonary metastasectomy from selected studies were listed in Table 3.

**Table 3:** **Summary of pulmonary metastasectomy from selected studies**

***Primary tumor type***	***Year***	***No. patients***	***5-year survival (%)***	***10-year survival (%)***	***References***
***Melanoma***	2007	1720	21	/	[40]
***Many types***	2011	575	46	/	[37]
***Colorectal carcinoma***	2002	165	39.6	37.2	[41]
***Colorectal carcinoma***	2007	175	53.8	20.6	[42]
***Renal cell carcinoma***	2002	191	41.5	/	[38]
***Renal cell carcinoma***	2011	202	39	/	[43]
***Testicular germ cell tumors***	1998	157	68	/	[44]
***Malignant fibrous histiocytoma***	2005	103	21	/	[45]
***Gynecologic cancers***	2006	103	46.8	34.3	[46]
**Bone sarcoma**	2010	52	31	/	[47]

SBRT has been shown to prolong OS or even induce a cure in lung oligometastases, which is especially attractive for patients who refuse or are unsuitable for resection. Results of retrospective studies have shown 2-year local control rates ranging 80-90% and 2-year OS rates ranging 66-84% [48-52]. These results were repeated in a 2010 systematic review involving 334 patients, in which the 2-year weighted local control rate was 77.9%, and the 2-year weighted OS rate was 53.7%. Recently, Takahashi et al. conducted a retrospective analysis of 42 patients with lung oligo-recurrence who underwent SBRT, 2-year local control rate and 2-year OS rate were 87% and 65% respectively [53]. Interestingly, the efficacy and feasibility of carbon ion radiotherapy for lung oligo-recurrence were evaluated in a retrospective study, 3-year local control rate was 85.4% and 3-year OS rate was 50.1%, without any grade 3–5 toxicity [54]. The safety and efficacy of SBRT for lung oligometastases has been confirmed by prospective phase I and II trials. In one multi-institutional phase I/II trial, use of SBRT at 48–60 Gy in 3 fractions showed no dose-limiting toxicities. In all, 60 Gy was delivered in phase II, and 1- and 2-year actuarial local control rates were 100% and 96%, respectively. The median survival was 19 months. Grade 3 toxicity occurred in 8% of patients with no grade 4 or higher toxicity [55]. Other prospective studies have shown similar results [56,57], which are comparable to survival after surgery. In summary, increasing evidence proves that SBRT is efficacious and well tolerated in patients with lung metastases. Summary of SBRT for lung metastasis from selected studies were listed in Table 4.

**Table 4:** **Summary of SBRT for lung metastasis from selected studies**

***Primary tumor type***	***Year***	***No. patients***	***Local control***	***Toxicity***	***References***
***Mixed***	2006	50	3-year local control rate 83%	35% Grade 1 toxicity, 6.1% grade 2 toxicity, 2% grade 3 toxicity	[51]
***Mixed (Most NSCLC)***	2009	124	3-year local control rate 83%	17.8% Grade 2 toxicity, 1.2% grade 3 toxicity	[48]
***Mixed (Most colorectal cancer)***	2010	10	1- and 2-year local control rates were 48% and 25%	Not mentioned	[58]
***Mixed (Most colorectal cancer)***	2011	44	1- and 2-year local control rates in colorectal cancers were 80% and 72%	2 cases of Grade 2 radiation pneumonitis, 1 case of Grade 3 pneumonitis, No Grades 4 radiation pneumonitis	[59]
***Mixed (Most NSCLC)***	2012	61	2- and 3- year local control rates were 89% and 83.5%	one case of grade 3 radiation pneumonitis	[52]
**Mixed (Most colorectal cancer)**	2013	32	1-, 2- and 3- year local control rates were 97%, 92% and 85%	no grade 4 toxicity, 3 cases of grade 3 toxicities, 1 case of grade 2 radiation pneumonitis	[60]

#### Adrenal oligometastases

Adrenal metastases, mostly lung and renal in origin, are reported to occur in 13-27% of disseminated malignancies at autopsy [61]. Although debates exist on adrenal metastasectomy, several retrospective studies have shown that both open and laparoscopic adrenalectomy prolongs survival [62,63]. In a 2012 retrospective study by Zheng *et al.*, 31/47 patients had adrenal metastasectomy, and survival rates were significantly higher in patients with surgery than in patients without surgery (34.2 ± 4.7 vs. 6.3 ± 2.7 months, respectively). Data on SBRT for treating adrenal metastases is limited. Several studies show that it is an invasive but effective and safe option [64,65]. Casamassima *et al.* studied 48 patients with adrenal metastases who were treated with SBRT at 36 Gy/3 fractions. Actuarial 1- and 2-year local control rates were 90%, and actuarial OS rates at 1 and 2 years were 39.7% and 14.5%, respectively.

#### Spinal oligometastases

Bone metastases account for 20% of patients with metastatic tumors, and the most common site is the axial skeleton. Spinal metastases often cause pain and diminished quality of life. Palliative surgery benefit patients with spinal metastasis by improving quality of life [66,67]. SBRT has emerged as a novel, promising and non-invasive treatment modality for isolated spinal metastasis [68,69]. In a phase I/II trail, 149 patients with 166 non-cord compressing spinal metastases were treated with 27–30 Gy SBRT, generally in 3 fractions. Actuarial survival rates at 1 and 2 years were 71.9% and 48.8%, respectively. The actuarial local control rates at 1 and 2 years after SBRT were 80.5% and 72.4%, respectively. Few grade 3 and no grade 4 or higher toxicities were observed. Compared to surgery, SBRT may better benefit patients with prolong survival, pain relief and safety. However, it should be noted that SBRT is associated with vertebral compression fractures [70,71].

#### Long-term follow-up data of oligometastases treated by SBRT

Although there are no prospective randomized studies comparing the long-term survival of SBRT and metastasectomy, the recent prospective study by Widder *et al.* found that the long-term survival of patients with pulmonary oligometastases treated with SBRT is not inferior to that of metastasectomy. In this study, the 5-year OS rates were 41% for metastasectomy and 49% for SBRT (p =0.43) [72].

Several studies have shown better prognosis in long-term survivors who had been treated with SBRT. Recently, de Vin *et al.* retrospectively studied 309 oligometastatic cancer patients treated with SBRT. They showed a 5-year OS rate of 32%, and they identified a subgroup of long-term survivors with a median survival of 40 (24–63) months [73]. Moreover, a prospective study by Milano et al. of 121 patients with oligometastases treated with SBRT showed a 6-year OS rate of 20%, and they identified selected long-term breast cancer survivors with a 6-year OS rate of 47% [74]. Clearly, the key issue for prospective randomized trials to verify the promising benefit of SBRT in patients with oligometastases will be how to identify the most suitable population for radical local treatment.

### Clinical factors in selecting patients with oligometastases

The satisfactory outcomes of local therapy for oligometastases strongly suggest the existence of an oligometastatic phenotype, but the frequency of oligometastases in various cancer types is unknown, and there are no standard criteria used to identify these patients. However, several clinical prognostic factors have been identified which strongly suggest the oligometastatic state and aid in selecting patients for aggressive local therapies.

#### Colorectal cancer

Liver is the most common site of metastasis from colorectal cancer, and more than half of patients with colorectal cancer will develop liver metastases during the disease course. As early as the 1980s, a subpopulation of patients with isolated liver metastases and a better prognosis was identified [75], and hepatic resection has become the standard treatment for resectable liver metastases from colorectal cancer. Recent data on the efficacy of this surgical resection has demonstrated a 5-year OS rate of 39-47% and a 10-year OS rate of 17-28%, which is far better than for those who received systemic therapy [76,77]. These long-term survivors strongly support the biological distinction of oligometastases in colorectal cancer. Simmonds *et al.* performed a review of 529 independent studies evaluating the benefit of hepatic resection of oligometastases from colorectal cancer and found a majority of retrospective studies and an absence of prospective randomized trials [78], indicating the need for further studies of this population.

Approximately 10-15% of patients with colorectal cancer will develop lung metastases, and pulmonary metastasectomy is the standard treatment for resectable lesions. Evidence suggests that patients with isolated lung metastases have a better prognosis after resection, with a 5-year OS rate of 40-61% [79,80]. Salah *et al.* reviewed 8 retrospective studies of pulmonary metastasectomy in 988 colorectal cancer patients, and the 5-year OS rate was 54.3% following the first lung resection [81]. Moreover, after further stratification into the population into good, intermediate, and high-risk groups according to three independent prognostic factors (CEA, disease free interval and lesion numbers), the 5-year OS were 68.2%, 46.4% and 26.1%, respectively; in this, the good and intermediate groups had OS comparable to patients with stage III colorectal cancer.

Although there is a lack of randomized controlled data on local therapy for metastases from colorectal cancer, the promising long-term survival suggests a potential cure in the oligometastatic population, while this survival is rare in patients with extensive metastases.

#### Breast cancer

The survival benefit of pulmonary metastasectomy for patients with breast cancer has been reported in retrospective studies, but until recently, no prospective randomized trials have been available to validate the existence of oligometastases in breast cancer and to explore the impact of local therapy. Recently, Meimarakis *et al.* studied the role of metastasectomy in breast cancer patients with oligometastases, at least partially [82]. They reported 5- and 10-year OS rates of 59.6% and 43.0%, respectively, which were significantly higher than those of patients treated regularly.

Liver metastases of breast cancer are usually part of generalized metastases and indicate a poor prognosis with a median OS of 4–12 months, but selected (<5%) patients with isolated liver metastases have shown good long-term survival after metastasectomy of liver lesions. A systematic review by Bergenfeldt *et al.* analyzed patients with breast cancer and liver oligometastases who had undergone metastasectomy and patients who had received local ablation therapy such as SBRT, they showed that surgical resection led to 5-year OS rates of 25-42%, that local ablation therapy had similar survival results, and R0 resection was a strong prognostic factor for survival [13].

#### Non-small cell lung cancer (NSCLC)

A systematic review by Ashworth et al. analyzed 49 retrospective studies of 2176 patients with NSCLC treated with metastasectomy and found highly variable survival outcomes, with 5-year OS rates of 10-80% and more than half of oligometastatic lesions progressing within 12 months after metastasectomy [83]. Interestingly, two randomized controlled trials, primarily of patients with NSCLC, have provided compelling evidence in support of an oligometastatic state. In 1990, Patchell *et al.* reported that patients with a single brain metastasis who had undergone metastasectomy plus radiotherapy had longer survival than those who had received radiotherapy alone [84]. In a randomized multi-institutional trial, 333 patients (64% NSCLC) with brain metastases received whole-brain radiation therapy with or without an SBRT boost; results for patients with a single brain metastases showed that the combination therapy group had a survival advantage compared to the whole-brain radiation group (median OS 6.5 vs 4.9 months, p =0.039) [85].

#### Other tumor origins

The different histology of tumors greatly influences prognosis, and this fact should be taken into account when determining patient treatment. Studies have shown that tumors from particular origins have a better prognosis after local therapy. Pulmonary metastases from germ cell tumors, hepatic metastases from neuroendocrine tumors (NET), and breast cancer oligometastases all have relatively prolonged survival, while hepatic metastasis from gastric cancer has a worse prognosis. A 2011 retrospective study of 575 patients and 708 lesions by Casiraghi *et al.* showed that tumor origin predicts prognosis after pulmonary metastasectomy, with 5-year survival rates of 46%, 39%, 37% and 90% for epithelial, sarcoma, melanoma and germ cell cancers, respectively.

#### Number of metastases in selecting patients with oligometastases

The number of metastatic lesions is known to influence prognosis, but the concrete number of lesions has not been determined. Several important studies confirmed that 4 metastases is a critical value. A systematic review of 15 studies by Spelt *et al.* demonstrated that number of metastases was a prognostic factor in all prospective studies and in 8/11 retrospective studies, and that number of metastases correlated best with survival [86].

#### DFI in selecting patients with oligometastases

DFI has generally been considered a prognostic factor in lung metastases other than liver metastases. The value of DFI has not been standardized, however, and varies between 12, 23 and 36 months in different studies. Kanzaki *et al.* studied patients with pulmonary metastasectomy from renal cell carcinoma and demonstrated that a DFI ≥2 years was associated with a 5-year survival of 58% but that the survival of patients with DFI <2 years decreased to 26% [87]. DFI has also been found to be a prognostic factor after pulmonary local therapy. Pfannschmidt *et al.* demonstrated a 5-year survival rate of 24.7% in patients with DFI <23 months compared to 47% for those with DFI >23 months [33].

#### N stage in selecting patients with oligometastases

Involvement of lymph nodes, also termed N staging, is a risk factor in several tumor types, particularly in liver metastases other than pulmonary metastases. Negative lymph node status predicts a better prognosis than positive status after local therapy. In a study of 925 patients by Rees *et al.* to evaluate factors associated with prognosis after liver metastasectomy for colorectal cancer, primary lymph node status was identified as a prognostic factor, and 5-year survival was 42.2% with lymph node-negative status and 31.8% with lymph-node-positive status [88].

#### Scoring systems in selecting patients with oligometastases

Scoring systems, which aim to predict prognosis before local therapy, are useful because individual patients have different prognostic factors. Myrddin *et al.* performed a prospective study of 929 patients to identify prognostic factors after hepatic resection for metastatic colorectal cancer [88], and the Basingstoke Predictive Index was created based on the following prognostic factors: primary lymph node status, primary tumor differentiation, CEA level at hepatectomy, metastatic number, largest tumor diameter and extrahepatic metastatic disease. Patients were given a total score based on the prognostic factors and were divided into the following groups: 0, 5, 10, 15, 20, 25, and 30. Five-year survival in patients with the lowest scores was 66%, decreasing to only 2% in patients with the highest scores. Similar results have been observed in other scoring systems, including Risk score, the Memorial Sloan Kettering Cancer Center clinical risk score and simplified cancer staging systems [89-91].

#### MiroRNA signature in selecting patients with oligometastases

MicroRNA is known to regulate proliferation and apoptosis in cancer development, and microRNA expression profiles may be better able to classify cancer subtypes than coding gene profiles. Emerging evidence indicates that microRNA profiles could be a useful tool to distinguish oligometastasis from polymetastasis. Recently, Lussier *et al.* analyzed microRNA expression patterns from lung metastasis samples of patients with 1–5 metastatic tumors resected with curative intent [92] and identified distinct microRNA profiles between patients with the highest and lowest rates of recurrence. Another clinical/pathologic correlation study indicated that microRNA-200c expression was able accurately to characterize patients with clinically limited metastases into two phenotypes-those whose disease progressed to widespread, polymetastatic recurrence and those with clinically limited disease or oligometastatic recurrence [92].

## Conclusions

Given the abundant clinical and biological evidence supporting the oligometastatic state, we infer that oligometastasis is more than a mirage. As understanding of the biology of metastases increases, improved biomarkers to accurately identify patients with oligometastases will be generated, allowing better selection for locally ablative therapies. Furthermore, prospective trials will be needed to clarify the criteria for selecting patients with oligometastases from each specific cancer type, and randomized trials comparing the efficacy and safety of different treatment modalities including SBRT or surgery will also be needed to generate guidelines for the treatment of oligometastasis. With the advance of molecular and clinical medicine, the oligometastasis mirage might be converted to a paradigm of cancer treatment.

## Abbreviations

SBRT: Stereotactic body radiotherapy
OS: Overall survival
NET: Neuroendocrine tumors
PFS: Progression-free survival
DFI: Disease-free interval
NSCLC: Non-small cell lung cancer
